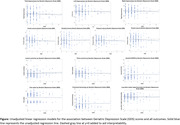# Gender differences in the association of depressive symptoms with neuroimaging outcomes in the *LifeAfter90* Study

**DOI:** 10.1002/alz.091828

**Published:** 2025-01-03

**Authors:** Alexander Ivan B. Posis, Yi Lor, Kristen M. George, Paola Gilsanz, Pauline Maillard, Charles Decarli, Claudia H. Kawas, María M. M. Corrada, Rachel A. Whitmer

**Affiliations:** ^1^ University of California, Davis, Davis, CA USA; ^2^ Kaiser Permanente Northern California Division of Research, Oakland, CA USA; ^3^ University of California, Irvine, Irvine, CA USA

## Abstract

**Background:**

Depressive symptoms are associated with reduced brain integrity. However, it is unclear if this association holds among a racially and ethnically diverse population aged ≥90 years and if there are differences by gender.

**Method:**

The *LifeAfter90* Study enrolled 212 long‐term members of Kaiser Permanente Northern California who were ≥90 years old, without dementia diagnosis, and received neuroimaging via 3T MRI. Depressive symptoms were measured using the 15‐item Geriatric Depression Scale (GDS). Neurodegeneration outcomes included volumetric measures (residual values accounting for total cranial volume) of total and bilateral hippocampus, regional cortical volume, and lateral and third ventricular volume, as well as amyloid PET, quantified as mean SUVR and amyloid positivity. Vascular injury outcomes included fractional anisotropy, free water, and log‐transformed total white matter hyperintensities. We used multivariable linear or logistic regression models to estimate associations between GDS and MRI outcomes adjusted for age at neuroimaging scan, gender, race/ethnicity, and education. We assessed effect measure modification by testing a gender‐by‐GDS interaction and gender‐stratified models.

**Result:**

Participants had a mean age of 93.1±2.2 years, 58% were women, 22% African American/Black, 27% Asian, 17% Hispanic/Latino, 28% White, and 5% Other/Multiracial. Average GDS score was 2.1±2.0 (women = 2.1±2.1; men = 2.1±1.9), and 16% had scores ≥4 (range = 0‐11), which suggests significant depressive symptoms. While increased GDS scores generally trended with poorer neuroimaging outcomes (e.g., β_Frontal Cortex_ = ‐0.29, 95% CI ‐0.88,0.30; except for occipital cortex volume, third ventricle volume, or vascular injury outcomes), associations were not significant in unadjusted (Figure) or multivariable models (p’s>0.05). There were differences by gender for third ventricle volume (p‐interaction = 0.01), such that increasing GDS scores were associated with lower third ventricle volumes among men only (β = ‐0.06; 95% CI ‐0.11,‐0.01). Increased GDS was associated with higher lateral ventricle volume among women only (β = 1.24; 95% CI 0.01,2.46), however the gender‐by‐GDS interaction was not significant (p‐interaction = 0.13).

**Conclusion:**

In this study of diverse adults aged 90+, increased depressive symptoms were associated with lower third ventricle volume among men and higher lateral ventricle volume among women. Results support future longitudinal study to understand change in neuroimaging outcomes that may be impacted by depression and gender among the oldest‐old.